# Intensive Care of a Weil's Disease With Multiorgan Failure

**DOI:** 10.4021/jocmr2010.05.302w

**Published:** 2010-06-15

**Authors:** Turkan Togal, Ali Sener, Neslihan Yucel, Semra Demirbilek, Feride Sinem Akgun, Mustafa Aydogan, Muharrem Ucar, M. Ozcan Ersoy

**Affiliations:** a Inonu University, School of Medicine, Department of Anesthesiology, Malatya, Turkey; b Inonu University, School of Medicine, Department of Emergency, Malatya, Turkey

## Abstract

Leptospirosis is a commonly encountered type of zoonosis, especially in tropical regions. There is insufficient data regarding its frequency in non-tropical regions such as Turkey. Although leptospirosis presents with a mild icteric form in nearly 90% of cases, it can lead to Weils disease characterized by fever as well as fulminant hepatorenal and respiratory failure, in approximately 5 - 10% of cases. In this case report, we present a patient with Weil's disease, complicated with multiorgan failure.

**Keywords:**

Weil's disease; Leptospirosis; Multiorgan failure

## Introduction

Leptospirosis is a re-emerging spirochetal zoonosis with a worldwide distribution affecting both animals and humans. The incidence of Leptospirosis is significantly high in regions with a warmer climate as compared to those with a temperate climate. The data regarding its frequency in non-tropical regions, such as Turkey, are insufficient [1]. Human infection results from exposure to infected urine of carrier mammals, either directly or via contamination of soil or water. The clinical syndromes may vary from a subclinical infection to a severe illness. The disease usually presents as a flu-like illness with mild hepatic and renal impairment. Severe forms of leptospirosis are characterized by severe hepatorenal dysfunction, mental status changes and haemorrhagic diathesis, but rarely with multiorgan dysfunction. Although it may potentially have a fulminant and fatal course, leptospirosis usually remains as an underdiagnosed cause of multiorgan failure. Pulmonary involvement is not uncommon (20 - 70%), but symptoms are usually mild without sequelae. Acute respiratory distress syndrome (ARDS) requiring artificial ventilation is very rare and has a high mortality rate of up to 50%, often associated with pulmonary haemorrhage due to endothelial damage.

Although leptospirosis presents with a mild icteric form in nearly 90% of cases, it can lead to Weil's disease characterized by fever as well as fulminant hepatorenal and respiratory failure in approximately 5 - 10% of cases. Weil's disease may potentially have a fulminant and fatal course and, therefore, requires critical care. Diagnosis based on clinical assessment alone may not always be accurate, yet the exact diagnosis may only be established upon clinical suspicion. The establishment of the diagnosis requires the presence of clinical symptomatology together with the demonstration of leptospirosis in blood or urine cultures, or on serological testing. The prompt initiation of antibiotherapy is key to disease control as well as the prevention of the urinary spread of the microorganism [2].

## Case Report

A 24-year-old male was admitted to the Department of Infectious Disease of the State Hospital with fever, lethargy, muscle pain, headache, productive cough, and decreased appetite. Upon evaluation he was put on ceftriaxon sodium, 2 g/day with suspicion diagnosis of meningitis. He was referred to Emergency Department of the University Hospital in the same province two days after admission since he failed to improve clinically despite an antibiotherapy. He lived on his own, smoked one and a half packs of cigar per day, and worked for the sewerage company. He had no significant past medical or surgical history and had not recently travelled outside the city. On admission, he was apathic and stuporous. Initial examination revealed neck stiffness and jaundice. His body temperature was 36.0^o^C, heart rate 122 beats/min, arterial blood pressure 90/60 mm Hg, respiratory rate 19/min and oxygen saturation 96% while breathing room air. On auscultation, his heart sounds were normal and air entry was equal on both lungs with occasional scattered wheezing. Initial laboratory investigations showed in Table 1. ECG showed sinus tachycardia and chest X-ray revealed mildly increased bronchoalveolar markings. An initial diagnosis of sepsis resulting from a lower respiratory tract infection, Weils disease, acute hepatitis, and acute renal impairment was made and he was transferred to a critical care ward. Within a few hours, his condition deteriorated. He was breathless, hypoxic, and hypotensive (arterial pressure 80/40 mmHg) despite the infusion of colloids and crystalloids. He became exhausted, deeply hypoxic, and acidotic (blood gas, FIO_2_ 0.9, pH 7.34, PaCO_2_ 4.8 kPa, base excess 26.4 mmol litre^-1^, bicarbonate 19.4 mmol litre^-1^, PaO_2_ 7.7 kPa, SaO_2_ 84%). Noradrenaline infusion was started to maintain a mean arterial pressure (MAP) of more than 70 mm Hg. Since his condition continued to rapidly deteriorate, the decision was made for tracheal intubation. Bilevel continuous positive airway pressure (BiPAP) and invasive monitoring was initiated. Investigations repeated. Repeat investigation revealed an extensive bilateral alveolar shadowing, stimulating ARDS as well as worsening of renal and hepatic function. Repeat laboratory investigations showed in Table 1. Abdominal ultrasound revealed no abnormalities, and a cardiac echo showed normal right and left ventricular function. With the background history of working for the sewerage company, along with hepatic and renal impairment, leptospirosis was suspected and appropriate samples were obtained and sent to the reference laboratory. Samples were also forward for atypical screening. Treatment was initiated with amoxicillin-sulbactam and meropenem, and later changed to teicoplanin and netilmycin, followed by tigecycline. Platelets, packs of erytrocytes and packs of fresh frozen plasma were transfused. Pressure control ventilation (PEEP 10 cmH_2_O, peak airway pressure 27 cmH_2_O, FIO_2_ 0.9) was instituted, maintaining a PaO_2_ of 90 mm Hg. High-dose noradrenaline (0.8 mgm kg^-1^ min^-1^) was required to maintain a MAP of 70 mm Hg. He had clinically adequate filling pressure and was started on a diuretic infusion, to which he responded well. There was significant pulmonary bleeding for the first 4 days and his platelets were low. He required sedation, and ventilatory support with lung protection strategy for 7 days before sedation and noradrenaline could be weaned off. Chest X-rays were compatible with ARDS and started to improve after one week. A percutaneous tracheostomy was performed on 10th day, using the method of Griggs. He required intermittent hemodialysis for 20 days, to be followed by bedside renal replacement therapy with hemodiafiltration (Prisma®). Later on he was weaned slowly after 10 days and was discharged to a medical ward on day 36. C-reactive Protein was down to 17.1. Although the initial testing for leptospirosis was negative, a second specimen was sent after 30 days, which resulted also negative for leptospirosis.

**Table 1:** Laboratory Findings During Management of Case

	First (Initial)	Second (Ten Days After)	Third (Before Discharge)
Leukocyte (/mm^3^)	37000	26900	7800
Hemoglobin (g/dl)	17.9	9.9	15
Hematocrit (%)	51	28.9	35
Platelet (/mm^3^)	36000	56000	206000
CRP (g/dl)	107	17.1	10.4
Glucose	43	110	135
LDH (U/L)	29		
ALP U/L	158		
Urea (mg/dl)	45	100	14
Creatinine (mg/dl)	4.18	4	1
AST (U/L)	> 4202 U/L	1041	37
ALT (U/L)	> 4113 U/L	1050	67
GGT (U/L)	82		30.5
Albumin	4		3.5
Sodium (mEq/lt)	142	145	143
Potassium (mEq/lt)	4.2	5.7	4
Total bilurubin (mg/dl)	4.3		1.5
Direct biluribin (mg/dl)	2.1		0.5
Protrombin time sec	26.7		
aPTT sec (24 - 36)	6.89		
Pt	16%		
Fibrinogen mg/dl	62.8		200
D-Dimer mikrogFE U/ml	1.36		
INR	2.48	1.17	1.1
Sedimentation	45		2
AntiHBsAg IU/ml (micro)	19.5		
Anti-HCV	Negative		
pH	7.386		7.35
pCO_2_ mm Hg	30		45
pO_2_ mm Hg	63		96
Saturation (%)	92.2		98
BE	-5.6		3

## Discussion

Leptospirosis has a wide clinical spectrum ranging from asymptomatic cases to severe Weils disease. Pathogenic mechanisms of leptospirosis may be divided into direct effects by Leptospira and host immune response to infection. One mechanism of virulence is motility and the ability of Leptospira to swim through viscous media. Motility is probably more important in initial infection and in dissemination of organisms from the site of entry to sites of end-organ damage such as lung, liver, kidney, eye, and brain. Severe forms show multiorgan involvement such as liver, kidney and lung. Diagnosis of leptospirosis can also be defined in accordance with WHO recommendations [3], where a diagnostic ‘score’ is obtained, based on six clinical criteria, two laboratory-determined criteria and an epidemiological criterion. Leptospirosis is suspected when the score is 20, with a strong presumption when the score is 24. Prognostic factors associated with mortality have only been evaluated in a few studies. In a Polynesian hospital, 71 severe cases of leptospirosis were studied retrospectively during a period of 2 years. All 71 patients were admitted to the intensive care unit. On multivariate analysis, three independent variables were found to be associated significantly with severity, namely oliguria, hypotension and abnormal chest auscultation (the presence of crackles or rhonchi). These symptoms are consistent with those reported in other series [4]. The mortality rate of 7% was within the range of 4 - 10% reported in studies from France and the USA [5, 6]. Differences in the mortality rate can be attributed to the criteria chosen for patient inclusion and to the severity of the disease.

In the presence of leptospirosis, survival depends on rapid diagnosis as well as early and appropriate management. The mortality rate for oliguric patients with acute renal failure is higher than that for patients with persistent diuresis [7, 8] 

The incidence of pulmonary involvement in leptospirosis varies between 17% and 70%. It was shown that the mortality rate rose from 4.8% to 11% when patients developed respiratory symptoms and ARDS. Cardiac involvement is probably more common than that reported [9]. With the background history of working for the sewerage company along with hepatic and renal failure, laboratory investigations revealed multiorgan failure in the patient presented hereby. He had disseminated intravascular coagulation and bleeding from multiple sites. He required intermittent hemodialysis for 20 days, followed by bedside renal replacement therapy with hemodiafiltration (Prisma®). He was weaned slowly and discharged to a medical ward on 36th day. Hepatic function and coagulation parameters normalized in 10 days, urea and creatinine levels remained high until 20 days of admission.

Functional or structural hepatic damage has been known to follow systemic infections, in one of two scenarios. In the first scenario, the infection primarily involves the liver, as is the case in spirochete, rickettsia, viral and myocobacterial infections. In the second scenario, intrahepatic cholestasis is observed in the absence of hepatic involvement [10]. Various factors can contribute to renal impairment in Weils disease. These include hypovolemia, endotoxin-associated vasoconstriction, ischemia and acute tubular necrosis. Renal involvement is a major cause of mortality. The Leptospiras are found in the renal parenchyma and the tubular lumen [11]. In our case, the diagnosis of leptospirosis could be verified neither in cultures and serological testin nor using dark field microscopy. The background history and clinical progress was deemed adequate to establish the diagnosis and, therefore, appropriate antibiotics covering leptospirosis were initiated.

Leptospires are sensitive to most antimicrobial agents in vitro, but the importance of the in-vitro sensitivity testing on clinical outcome for these agents has not been assessed in clinical trials. A recent report shows that while Leptospira are sensitive in vitro to several antimicrobial classes, some variability was reported in the in-vitro susceptibility of various Leptospira species to a range of newer (ampicillin-sulbactam, cefotaxime, ceftriaxone, azithromycin, telithromycin, ciprofloxacin, moxifloxacin) and older antimicrobials (penicillin, ampicillin, amoxicillin, doxycycline, tetracycline, chloramphenicol, erythromycin). Many of the Leptospira species tested were more sensitive to ampicillin/sulbactam than to ampicillin alone [2]. Due to the observation of hospital infections caused by agents such as pseudomonas auroginosa, acinetobacter baumannii, candida albicans, that are among usual suspects in the setting of ICU and that are grown in serial cultures, treatment with antibiotics is continued in accordance with sensitivity testing. Impatient with the severe form of leptospirosis, clinical improvement has been reported to take two to four weeks. Pulmonary complications, massive bleeding in particular, are the cause of death in 20 to 70% of cases with leptospirosis [12]. In a study reporting on autopsy findings for 62 cases of leptospirosis in India, young males presenting with fever, dyspnea, hemoptysis, bleeding, oliguria and jaundice were observed to die immediately following their hospital admissions. Nad massive interalveolar hemorrhage (48 cases), acute interstitial nephritis or acute tubular necrosis (45 cases), and myocarditis (24 cases) were observed postmortem to support the serological diagnosis of leptospirosis. Wide spread bleeding was observed in the heart, gastrointestinal system, brain, pancreas and adrenal gland, yet the cause of death was pulmonary hemorrhage [13]. In our case, fresh frozen plasma as well as thrombocyte and erythrocyte suspensions were administered due to the presence of repeated hemorrhage from gastrointestinal system and lungs.

The presence of multiorgan failure necessitates the administration of appropriate antibiotherapy, circulatory and respiratory support, hemodialisis, hemodiafiltration, and replacement of blood product. In our patient, the reversal of multiorgan failure was observed by 20 days despite prompt administration of antibiotics and hemodialisis. Prolonged tracheal intubation required a tracheotomy although recovery was accelerated following the tracheotomy. Pulmonary complications have been reported to recover in the absence of sequlae under respiratory support [12]. In our case, the administration of antibiotic therapy, early supportive care, enteral nutrition and intensive blood replacement provided clinical improvement and the patient recovered completely. This case is reported in attempt to remind clinicians to consider leptospirosis in the evaluation of a patient with sepsis who develops multiple organ dysfunctions. Appropriate antimicrobial therapy can be life-saving.

## References

[1] Aydemir S, Ustundag Y, Borazan A, Sekitmez N, Ozdemir H (2004). A case with jaundice, acute renal failure and thrombocytopenia: Weil's disease. Academic Gastroenterology journal.

[2] Bharti AR, Nally JE, Ricaldi JN, Matthias MA, Diaz MM, Lovett MA, Levett PN (2003). Leptospirosis: a zoonotic disease of global importance. Lancet Infect Dis.

[3] Faine S (1982). Guidelines for the control of leptospirosis. Geneva: World Health Organization.

[4] Ko AI, Galvao Reis M, Ribeiro Dourado CM, Johnson WD, Riley LW (1999). Urban epidemic of severe leptospirosis in Brazil. Salvador Leptospirosis Study Group. Lancet.

[5] Bourrier P, Chennbault JM, Achard J (1988). Leptospiroses: analyse retrospective de 99 cas observe's en 10 ans dans le Centre-Ouest de la France. Me'd Mal Infect.

[6] Heath CW, Alexander AD, Galton MM (1965). Leptospirosis in the United States. Analysis of 483 cases in man, 1949, 1961. N Engl J Med.

[7] Abdulkader RC (1997). Acute renal failure in leptospirosis. Ren Fail.

[8] Magaldi AJ, Yasuda PN, Kudo LH, Seguro AC, Rocha AS (1992). Renal involvement in leptospirosis: a pathophysiologic study. Nephron.

[9] Doudier B, Garcia S, Quennee V, Jarno P, Brouqui P (2006). Prognostic factors associated with severe leptospirosis. Clin Microbiol Infect.

[10] Farrell GC, Feldman M, Scharschmidt BF, Sleisenger MH (1998). Hepatic manifestation of systemic disease and other disorders of the liver. Sleisenger and Fordtran's gastrontestinal and liver disease: Pathophysiology/ diagnosis/ management.

[11] Seguro AC, Lomar AV, Rocha AS (1990). Acute renal failure of leptospirosis: nonoliguric and hypokalemic forms. Nephron.

[12] Thammakumpee K, Silpapojakul K, Borrirak B (2005). Leptospirosis and its pulmonary complications. Respirology.

[13] Salkade HP, Divate S, Deshpande JR, Kawishwar V, Chaturvedi R, Kandalkar BM, Vaideeswar P (2005). A study of sutopsy findings in 62 cases of leptospirosis in a metropolitan city in India. J Postgrad Med.

